# Third-Generation Cephalosporin-Resistant Enterobacterales and Methicillin-Resistant *Staphylococcus aureus* (MRSA) in Pigs in Rwanda

**DOI:** 10.3390/ani16010122

**Published:** 2025-12-31

**Authors:** Emmanuel Irimaso, Valens Hagenimana, Emmanuel Nzabamwita, Michael Blümlinger, Otto W. Fischer, Lukas Schwarz, Michael P. Szostak, Olga Makarova, Adriana Cabal Rosel, Werner Ruppitsch, Elke Müller, Andrea T. Feßler, Sascha D. Braun, Stefan Schwarz, Stefan Monecke, Ralf Ehricht, Suzana Tkalcic, Christophe Ntakirutimana, Joachim Spergser, Doris Verhovsek, Igor Loncaric

**Affiliations:** 1 Institute of Microbiology, University of Veterinary Medicine, 1210 Vienna, Austria; e.irimaso@ur.ac.rw (E.I.); michael.bluemlinger@vetmeduni.ac.at (M.B.); michael.szostak@vetmeduni.ac.at (M.P.S.); joachim.spergser@vetmeduni.ac.at (J.S.); 2 The College of Veterinary Medicine and Animal Sciences (CVAS), University of Rwanda, Nyagatare P.O. Box 57, Rwanda; vhagenimana1997@gmail.com (V.H.); nzabemma123@gmail.com (E.N.); 3 Clinical Center for Population Medicine in Fish, Pig and Poultry, Clinical Department for Farm Animals and Food System Science, University of Veterinary Medicine, 1210 Vienna, Austria; lukas.schwarz@vetmeduni.ac.at (L.S.); doris.verhovsek@vetmeduni.ac.at (D.V.); 4 New Vision Veterinary Hospital (NVVH), RN 4 Kigali—Musanze Road, Musanze, Rwanda; owfischer@aol.com (O.W.F.); ntakirutabrian90@gmail.com (C.N.); 5 Centre for Food Science and Veterinary Public Health, Clinical Department for Farm Animals and Food System Science, University of Veterinary Medicine, 1210 Vienna, Austria; olga.makarova@vetmeduni.ac.at; 6 Institute for Surveillance & Infectious Disease Epidemiology, Division Public Health, Austrian Agency for Health and Food Safety, 1200 Vienna, Austria; adriana.cabal-rosel@ages.at; 7 Institute of Hygiene and Medical Microbiology, Medical University Innsbruck, 6020 Innsbruck, Austria; werner.ruppitsch@i-med.ac.at; 8 Faculty of Food Technology, Food Safety and Ecology, University of Donja Gorica, 81000 Podgorica, Montenegro; 9 Leibniz-Institute of Photonic Technology (Leibniz-IPHT), Member of the Leibniz Center for Photonics in Infection Research (LPI), 07745 Jena, Germany; elke.mueller@leibniz-ipht.de (E.M.); sascha.braun@leibniz-ipht.de (S.D.B.); stefan.monecke@leibniz-ipht.de (S.M.); ralf.ehricht@leibniz-ipht.de (R.E.); 10 InfectoGnostics Research Campus, 07743 Jena, Germany; 11 Center for Translational Medicine (CETRAMED), Jena University Hospital, Friedrich Schiller University Jena, 07747 Jena, Germany; 12 Institute of Microbiology and Epizootics, School of Veterinary Medicine, Freie Universität Berlin, Robert-von-Ostertag-Straße 7, 14163 Berlin, Germany; andrea.fessler@fu-berlin.de (A.T.F.); stefan.schwarz@fu-berlin.de (S.S.); 13 Veterinary Centre for Resistance Research (TZR), School of Veterinary Medicine, Freie Universität Berlin, Robert-von-Ostertag-Straße 8, 14163 Berlin, Germany; 14 Institute of Physical Chemistry, Friedrich Schiller University Jena, 07743 Jena, Germany; 15 College of Veterinary Medicine, Western University of Health Sciences, Pomona, CA 91766-1854, USA; stkalcic@westernu.edu

**Keywords:** *Staphylococcus aureus*, *E. coli*, *Klebsiella pneumoniae*, Enterobacterales, MRSA, ESBL, porcine, One Health approach, antimicrobial resistance

## Abstract

This pilot study investigated the presence of methicillin-resistant *Staphylococcus aureus* (MRSA) and third-generation cephalosporin-resistant (3GC-R) Enterobacterales in conventionally kept domestic pigs and their environment across four districts in Rwanda. A total of 114 swabs (nasal, rectal, manure, dust) from 29 farms were collected and processed to isolate resistant bacteria. Thirty-two 3GC-R Enterobacterales were detected. *Escherichia coli* predominantly harboring *bla*_CTX-M_ group 1 β-lactamase genes, alongside *Klebsiella pneumoniae* isolates, all displaying extended-spectrum β-lactamase (ESBL) phenotypes. Four MRSA isolates, all belonging to clonal complex 398 and SCC*mec* type IV, the typical livestock MRSA, were recovered from nasal and environmental samples. Multidrug resistance was frequently observed. The co-occurrence of β-lactamase genes, non-β-lactam resistance genes, and virulence factors such as *fimH* and loci associated with extraintestinal pathogenic and enteropathogenic *E. coli*. The detection of both MRSA and 3GC-R Enterobacterales in the present study indicates pigs and their farm environments as reservoirs of WHO priority pathogens in Rwanda, highlighting a potential public health risk in the context of extensive human–animal–environment interaction. These findings emphasize the urgent need for integrated One Health surveillance and comprehensive AMR control strategies addressing both animal and environmental reservoirs to support Rwanda’s National Action Plan on Antimicrobial Resistance.

## Simple Summary

This study explored the presence of antibiotic-resistant bacteria in pigs and their surroundings on farms in Rwanda. Antibiotic resistance occurs when bacteria change and become harder to kill with medicines, which is a serious problem for both human and animal health worldwide. We collected samples from pigs and their environment, such as nasal swabs, feces, manure, and dust, to check for two types of resistant bacteria: one called MRSA, which can cause tough infections in people and animals, and another group that resists important antibiotics often used to treat infections. Resistant bacteria were especially common in pig droppings and nasal samples. The study highlights the risk of sharing such bacteria where people and animals live closely together, as is common in Rwanda. These findings show the need to carefully watch for and control antibiotic resistance in animals and their environment, not just in people. This work supports efforts to protect health by promoting safer farming practices and responsible use of antibiotics in Rwanda.

## 1. Introduction

Antimicrobial resistance (AMR) has been a critical One Health challenge for many years, as multidrug-resistant bacterial pathogens complicate disease management in both human and veterinary medicine [1]. This global problem is driven by factors such as poor sanitation, global travel and trade, genetic mutations, and horizontal gene transfer, but is primarily accelerated by the intensive and suboptimal use of antimicrobials in humans and livestock [2]. Consequently, the resulting dissemination of resistance across human, animl and environmental interfaces poses a substantial threat to public health.

In this regard, antimicrobial-resistant bacteria of most significant concern are identified by the World Health Organization (WHO). The WHO published the WHO Bacterial Priority Pathogens List (WHO BPPL) [3]. While well-documented data are available from Europe, America, and China [4,5,6,7], there is still a paucity of information about the presence of 3GC-R Enterobacterales and MRSA in pigs from Africa in general [8] and Rwanda in particular. This is of importance due to historical connections with Europe [9], wherefrom parent stock imports could introduce MRSA, particularly livestock-associated MRSA (LA-MRSA) belonging to clonal complex (CC) 398, which is predominant in Europe [10] as well as 3GC-R Enterobacterales, omnipresent among pigs in the EU [11]. In Rwanda, pig farming is experiencing rapid growth due to its role in ensuring food security and contributing to the country’s economic development. This growth is driven by increasing demand for pork in urban and rural areas and the potential for export to neighboring countries [12]. This rapid expansion poses a potential risk for AMR, as increased production often leads to higher antimicrobial usage to prevent and treat infections in dense animal populations, which can promote the emergence and spread of resistant bacteria [13].

**Figure 1:**
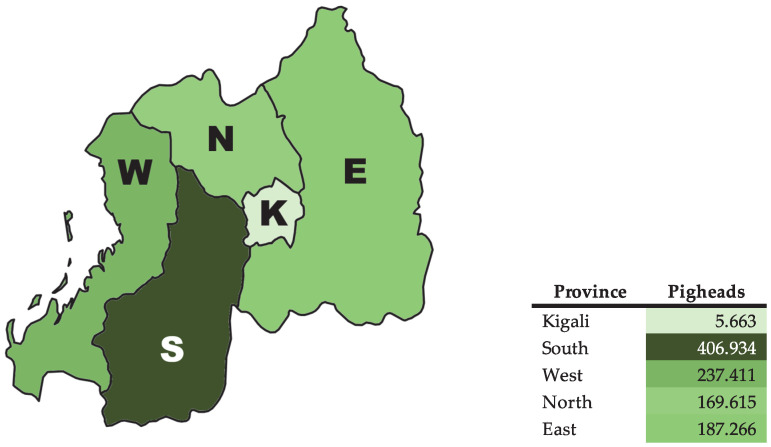
Pig density in Rwanda, W, West Province; N, North Province; E, East Province; K, Kigali; S, South Province.

In Rwanda, MRSA and cephalosporin-resistant Enterobacterales are acknowledged challenges within the Rwandan human healthcare sector [14,15,16,17,18,19,20,21]. However, currently, little is known about the occurrence of these pathogens in animals in Rwanda. Recently, our working group reported on the presence of 3GC-R Enterobacterales in ruminants [22] that share the same environment as pigs. The households in Rwanda usually raise different types of livestock. The highest number of pigheads is in South Province (406.934), followed by West Province (237.411), East Province (187.266), North Province (169.615), and Kigali (5663) (Figure 1) [23]. Pig farming in Rwanda is mainly characterized by small-scale production, where pigs are often housed near the owners’ homes and in close contact with other domestic animals, and in some regions of the country, 80% of households are estimated to keep pigs, with 1–2 grown pigs per household [12]. This close proximity between people and animals offers a valuable opportunity for antimicrobial resistance studies under the One Health approach. Therefore, the present study aims to investigate the presence of MRSA and 3GC-R Enterobacterales in conventionally kept domestic pigs and their environment in Rwanda.

## 2. Materials and Methods

### 2.1. Sample Collection and Isolation, and Identification of Third-Generation Cephalosporin-Resistant Enterobacterales and Methicillin-Resistant Staphylococcus aureus (MRSA) and Estimation of Confidence Intervals

A total of 114 swabs were collected during June 2023 (35 rectal, 27 nasal, and 52 environmental) from 29 farms across four districts of Rwanda (Musanze, North Province, n = 70; Nyagatare, East Province, *n* = 27; Bugesera, East Province, *n* = 9; Rwamagana, East Province, *n* = 8). Samples were collected from one pig (nasal, rectal) and one environmental sample (dust, manure) per farm. Farms were selected based on the owner’s willingness to participate in the study. Sample collection was performed under conditions suitable during sampling and with cooperation from farmers. The study was discussed, and the swabbing was approved by the Research Screening and Ethical Clearance Committee of the College of Agriculture, Animal Sciences and Veterinary Medicine, University of Rwanda (007/2023/DRI from 30 May 2023) in Nyagatare. Cultivation of bacteria was performed in a microbiological laboratory at the New Vision Veterinary Hospital (NVVH) in Musanze, Rwanda (https://nvvh.rw/). All samples were examined for the presence of 3GC-R Enterobacterales and MRSA. For the cultivation of 3GC-R Enterobacterales, swabs were first incubated overnight at 37 °C in buffered peptone water (Merck, Rahway, NJ, USA) with cefotaxime (1 mg/L) and subsequently cultured overnight at 37 °C on MacConkey agar (Oxoid; Basingstoke, UK) supplemented with cefotaxime (1 mg/L) (MacCTX). After incubation on MacCTX, one colony with a typical appearance characteristic for Enterobacterales [24] representing each distinct colonial morphology was subcultured on the same medium and then cryoconserved in a Thioglycollate medium (Beckton Dickinson (BD); Heidelberg, Germany) with 50% (*w*/*v*) glycerin: ratio 750:600 µL. For the isolation of MRSA, swabs were incubated overnight in tryptic soy broth (TSB) ((BD); Heidelberg, Germany) with 6.5% (*w*/*v*) NaCl and a 300 µL aliquot of each enriched TSB was cryoconserved at −25 °C, and together with presumptive 3GC-R Enterobacterales sent to the Institute of Microbiology, University of Veterinary Medicine, Vienna, Austria.

In Vienna, an aliquot of cryoconserved TSB was recultured in the same medium and then incubated on BBL™ CHROMagar™ MRSA II (BD; Heidelberg, Germany) for the isolation of MRSA. The *S. aureus* colonies that showed the typical colony pattern of MRSA on this medium were selected. The 3GC-R Enterobacterales were identified if the strain was resistant to Cefotaxime and Ceftazidime according to the CLSI standards [25]. Matrix-assisted laser desorption ionization time-of-flight mass spectrometry (MALDI-TOF MS) (Bruker Daltonik; Bremen, Germany) was used to identify all presumptive colonies to the species level. Only isolates confirmed as Enterobacterales and *S. aureus* were selected for further characterization.

### 2.2. Data Analysis

To determine the proportions of individuals and environment-tested 3GC-R Enterobacterales and MRSA positive per animal or environmental sample, 95% confidence intervals were estimated using the Clopper–Pearson exact method via the binom.test function in R [26].

### 2.3. Antimicrobial Susceptibility Testing

Antimicrobial susceptibility testing of Enterobacterales was performed by agar disk diffusion according to the CLSI standards [25]. *E. coli* ATCC^®^ 25922 served as the quality control strain. Disks containing the following antimicrobial agents were used: cefotaxime (30 μg); ceftazidime (30 μg); cefoxitin (30 μg); meropenem (10 μg); gentamicin (10 μg); tobramycin (10 μg); amikacin (30 μg); ciprofloxacin (5 μg); trimethoprim–sulfamethoxazole (1.25/23.75 μg); tetracycline (30 μg); chloramphenicol (30 μg); and nitrofurantoin (300 μg) (BD; Heidelberg, Germany). MRSA was confirmed by cefoxitin resistance [25]. Antimicrobial susceptibility testing of MRSA was performed with the following antimicrobial agents: gentamicin (GEN, 10 μg), erythromycin (ERY, 15 μg), penicillin (PEN, 10 IU), ciprofloxacin (CIP, 5 μg), clindamycin (CLI, 2 μg), tetracycline (TET, 30 μg), trimethoprim–sulfamethoxazole (SXT, 1.25/23.75 μg), chloramphenicol (CHL, 30 μg), and linezolid (LZD, 30 μg) (BD; Heidelberg, Germany. The reference strain *S. aureus* ATCC^®^ 25923 served as a quality control strain.

### 2.4. Molecular Characterization

Genomic DNA of Enterobacterales and MRSA was extracted after lysis enhancement by lysis enhancer and lysis buffer provided within INTER-ARRAY kits using the Qiagen DNeasy Blood & Tissue kit (Qiagen GmbH, Hilden, Germany) according to the manufacturer’s instructions. All samples were checked for DNA quantity and quality via a spectrophotometer (NanoDrop 2000 Spectrophotometer, Fisher Scientific (Austria) GmbH, Vienna, Austria) according to the manufacturer’s instructions. Resistance and virulence genes of Enterobacterales were analyzed by the INTER-ARRAY Genotyping Kit CarbaResist (INTER-ARRAY by fzmb GmbH; Bad Langensalza, Germany) [27] as well as by PCR (i.e., *tet*(A), *tet*(B)) as described elsewhere [28]. Detection and analysis of virulence-associated genes of *E. coli* isolates were performed using custom-made microarrays from INTER-ARRAY (INTER-ARRAY by fzmb GmbH, Bad Langensalza, Germany) according to the manufacturer’s instructions [29]. The phylogroup of the *E. coli* isolates was determined by the revised Clermont method [30]. Molecular characterization of MRSA was performed after DNA extraction using a DNA microarray-based technology (INTER-ARRAY Genotyping Kit *S. aureus*, Bad Langensalza, Germany), which is used for the detection of antimicrobial resistance and virulence-associated genes [31]. MRSA isolates were genotyped by *spa* typing. For *spa* typing, the polymorphic X-region of the protein A (*spa*) was amplified and sequenced according to the Ridom Spa Server protocol (https://spa.ridom.de/, accessed on 1 September 2025). *spa* types were determined using Ridom SeqSphere + Software v8.4 (Ridom, Münster, Germany).

## 3. Results

### 3.1. Presence of 3GC-R and MRSA

A total of 32 third-generation cephalosporin-resistant (3GC-R) Enterobacterales were detected, including 28 *Escherichia coli* isolates from 12 farms across all four districts. In addition, four *Klebsiella* (*K*.) *pneumoniae* isolates originated from two farms in Nyagatare district. Four methicillin-resistant *Staphylococcus aureus* (MRSA) isolates were recovered from two farms located in the Nyagatare and Bugesera districts. The prevalence of 3GC-R Enterobacterales was highest in rectal samples (60.0%; 95% CI: 42.1–76.1), followed by manure samples (18.5%; 95% CI: 6.3–38.1), nasal swabs (14.8%; 95% CI: 4.2–33.7), and dust samples (8.0%; 95% CI: 1.0–26.0). *E. coli* isolates were predominantly isolated from rectal swabs (*n* = 19), followed by manure swabs (*n* = 4), nasal swabs (*n* = 3), and dust swabs (*n* = 2). *K. pneumoniae* isolates were recovered from two rectal samples, one nasal swab, and one manure sample (Table 1). MRSA was detected in two manure samples, corresponding to 7.4% of manure samples (95% CI: 0.9–24.3), as well as in one nasal swab (3.7%; 95% CI: 0.1–19.0) and one dust sample (4.0%; 95% CI: 0.1–20.4).

### 3.2. Antimicrobial Susceptibility Testing and Characterization of Third-Generation Cephalosporin-Resistant (3GC-R) Enterobacterales

All Enterobacterales were susceptible to meropenem, amikacin, gentamicin, and nitrofurantoin, and displayed an ESBL phenotype, of which one isolate displayed an ESBL and an AmpC phenotype as well. The most commonly observed non-β-lactam resistance was against combined tetracycline and trimethoprim–sulfamethoxazole (*n* = 14 in *E. coli* and in all *K. pneumoniae*), followed by only tetracycline (*n* = 5) (Table 1). Eighteen out of 28 *E. coli* and all *K. pneumoniae* isolates were multidrug-resistant. Various resistance genes were detected. Among genes coding for β-lactamases, the *bla*_CTX-M_ gene family was detected in all Enterobacterales, with *bla*_CTX-M-1/15_ being predominant (*n* = 26 in *E. coli*), followed by *bla*_CTX-M9_ (*n* = 2 in *E. coli*). The *bla*_TEM_ genes were detected in 17 *E. coli* isolates, and *bla*_OXA-1_, *bla*_CMY_, and *bla*_ACT_ were only present in single isolates. All *K. pneumoniae* isolates carried *bla*_CTX-M-1/15_, *bla*_TEM_, and *bla*_SHV_ (Table 1). Various non-β-lactam resistance genes were detected in *E. coli* (*tet*(A), *tet*(B), *aac(6′)-Ib*, *aadA1*, *aadA2*, *aadA4*, *qnrS*, *sul1*, *sul2*, *dfrA5*, *dfrA12*, *dfrA13*, *dfrA14*, *dfrA17*, and *dfrA19*). In all *K. pneumoniae*, the resistance genes *tet*(A), *qnrB, qnrS, sul2*, and *dfrA14* were observed (Table 1). The most common *E. coli* virulence genes determined via microarray were *fimH*, which was detected in all isolates. In addition to the *fimH* gene, *astA*, *papC*, and *iucD* (*n* = 1), *papC* and *iucD* (*n* = 1), and *eae*, *acfC*, *escV*, and *espL* (*n* = 1) were found (Table 1).

The most common *E. coli* phylogenetic group was A (*n* = 17). Ten *E. coli* isolates belonged to phylogroup B1, with one isolate representing phylogroup E (Table 1).

### 3.3. Phenotypic and Genetic Profiling of MRSA Strains

All four MRSA isolates were resistant to gentamicin and tetracycline, which is well reflected by the observation that these isolates carried the resistance genes *aacA-aphD* and *tet*(M). All four MRSA isolates detected belonged to SCC*mec* type IV, *spa* t011, and clonal complex (CC) 398. Virulence-associated genes *lukF*, *lukS, lukX*, *lukY*, *hlgA*, *hla*, *hlb*, and *hld* were detected (Table 2 and Supplementary Table S1).

## 4. Discussion

An integrated One Health approach is necessary to estimate 3GC-R Enterobacterales and MRSA hazards, demanding simultaneous studies in humans, animals, and the environment. To our knowledge, this study is the first to demonstrate the combined presence of MRSA and 3GC-R Enterobacterales in pigs and their environment in Rwanda.

Very recently, Geuther et al. [17] investigated the presence of extended-spectrum beta-lactamase (ESBL)-producing Enterobacterales in various samples from humans, livestock, including pigs, environmental sources (soil, water, vegetables), and animal products in community households of Sovu, Southern Rwanda. Although Geuther et al. did not perform molecular characterization of ESBL-positive Enterobacterales, nor did they provide specific animal data, their findings remain important. A relatively high proportion of samples in that study were positive, highest for humans (37.9%) and livestock (15.6%). In our previous study on ruminants from Rwanda, we reported an overall prevalence of 12.8% (95% CI: 9.8–16.2), with prevalences of 16.3% in bovines (95% CI: 11.5–22.1), 11.8% in caprines (95% CI: 7.3–17.6), and 6.2% in ovines (95% CI: 2.0–13.8). These values are lower than those observed in pigs in the present study. In the present study, the majority of *E. coli* and all *K. pneumoniae* isolates carried genes for the CTX-M Group 1 (*bla*_CTX-M1/15_). β-lactamases of this group, especially *bla*_CTX-M-15_, were also the predominant enzymes detected in two previous Rwandan studies on 3GC-R Enterobacterales from humans, bovines, ovines, and caprines [18,22]. Globally, these enzymes are the primary mediators of third-generation cephalosporin resistance and are carried on highly mobile genetic elements [32]. The co-detection of antimicrobial resistance genes and various pathotype-specific virulence genes, albeit in three *E. coli* isolates, is an important observation. The *fimH* gene, encoding the Type 1 fimbrial adhesin, was present in all isolates; additionally, *papC* (P fimbriae) and *iucD* (aerobactin) were detected in two strains. These genes are classic markers of extraintestinal pathogenic *E. coli* (ExPEC) and their subgroup, Uropathogenic *E. coli* (UPEC) [33]. In addition, the presence of the Locus of Enterocyte Effacement (LEE) genes (*eae*, *acfC*, *escV*, and *espL*) in a single isolate is significant, indicating the presence of a strain that might be associated with Enteropathogenic *E. coli* (EPEC) or Enterohemorrhagic *E. coli* (EHEC) [34].

In contrast to 3GC-R Enterobacterales, MRSA was rarely detected. Typically, MRSA colonization in pigs is assessed via nasal swabs, while porcine 3GC-R Enterobacterales are investigated from rectal swabs [35]. In the present study, all sample types were examined concurrently. In some European countries, like Germany, Spain, and the Netherlands, nasal MRSA colonization of pigs is reported to be more than >50.0% [36]. The presence of MRSA isolates with indistinguishable characteristics on two farms in the present study may suggest an exchange of these isolates between these two farms. All MRSA belonged to CC398 and carried SCC*mec* type IV. To our knowledge, this is the first report of MRSA isolates from animals in Rwanda, specifically livestock-associated MRSA (LA-MRSA). The CC398-MRSA carrying SCC*mec* IV (CC398-MRSA-IV) is a relatively uncommon LA-MRSA among pigs [37] and is frequently detected in companion animals and horses [36,37,38,39]. In general, the detection of MRSA among animals expands the known scope of the Rwandan antimicrobial resistance (AMR) issue. Prior to the detection of MRSA in the framework of this study, MRSA was recovered from clinical specimens in humans in the Southern Province, Huye District, as well as among pediatric patients in Kigali [14,15]. MRSA that originated from the Huye District was molecularly characterized by SCC*mec* typing, detecting SCC*mec* type I and SCC*mec* type IV, respectively, with 26.6% MRSA isolates remaining non-typeable, mainly due to the limited discriminatory power of the method used [15]. No molecular characterization was conducted for isolates detected in Kigali [14]. During a study characterizing staphylococci from neonatal blood cultures in low- and middle-income countries, a single MRSA isolate from Rwanda underwent whole genome sequencing and was identified as sequence type ST22 within clonal complex CC22 [40].

Although this human data on MRSA and those on 3GC-R Enterobacterales in Rwanda were limited, initial findings from human medicine were sufficient for Rwanda to recognize the escalating threat of the AMR crisis across both human and animal health, mirroring concerns in other low- and middle-income countries. As a result, based on five objectives of the Global Action Plan of the World Health Organization (WHO), Rwanda developed the National Action Plan on Antimicrobial Resistance (NAPAMR) 2020–2024 that was extended for another five years (2025–2029) [41].

The present study showed that pigs and the pig farm environment in Rwanda may be a reservoir of bacteria that are listed as priority pathogens by WHO, either from the Critical group or High group. Pig farms are widely recognized as reservoirs for both MRSA and 3GC-R Enterobacterales [35]. This could be of critical importance for public health due to the small-scale, close-contact farming system prevalent in Rwanda, where pig housing is often near human residences and other domestic animals [12].

The present study, while providing novel insights into AMR in Rwandan pig farms, has limitations that need to be taken into consideration. Our pilot study has several limitations, from logistical and operational challe nges inherent to resource-limited settings, such as difficult weather and very restricted farm accessibility. The small geographical scope (four districts) and convenience-based sampling (dependent on farmer willingness) limit the representativeness of the findings for the entire Rwandan pig sector. We collected only one pig sample and one environmental sample (manure or dust) per farm, which may result in a potential sampling bias in the occurrence estimates. Furthermore, the cross-sectional design and single-sample-per-farm approach prevent a detailed elucidation of transmission dynamics. Finally, the exclusion of human specimens and other domestic animals, coupled with a DNA-array panel that did not cover all potential resistance genes, constrained our ability to better characterize the farm-level resistome and inter-species transmission.

## 5. Conclusions

The present study identified pigs and their environments as reservoirs for WHO priority pathogens in Rwanda, specifically detecting 3GC-R Enterobacterales dominated by *bla*_CTX-M_-positive *E. coli* and MRSA belonging to CC398. The presence of multidrug resistance and virulence factors in these isolates underscores a public health risk, highlighting the urgent need for integrated One Health surveillance in the region. The persistence of MRSA and 3GC-R Enterobacterales on pig farms necessitates a comprehensive understanding of all transmission routes. Investigations should include environmental vectors such as flies [42] and rodents [43,44], both of which are recognized carriers of these resistant pathogens. Consequently, effective hygiene protocols must extend beyond livestock management to address these important environmental reservoirs. Future AMR surveillance and control strategies must expand beyond direct animal sampling to prioritize understanding and mitigating transmission pathways involving the farm environment, as well as mobile vectors, to effectively support the goals of the Rwandan National Action Plan on Antimicrobial Resistance (NAPAMR).

## Figures and Tables

**Table 1:** Phenotypic and genotypic characteristics of third-generation cephalosporin-resistant Enterobacterales.

Strain ID	Origin *	Farm	District	Species	PG **	Non-ß-Lactam Resistance Phenotype ***	Resistance Genotype	VAG ****
N176 P3Ma	N	c	Musanze	*E. coli*	E	TET, SXT	*bla*_CTX-M1/15_, *bla*_TEM_, *tet*(A), *tet*(B), *qnrS*, *sul2*, *dfrA14*	*fimH*
N176 P3MDb	D	c	Musanze	*E. coli*	A	TET, SXT	*bla*_CTX-M1/15_, *bla*_TEM_, *tet*(A), *tet*(B), *qnrS*, *sul2*, *dfrA15*	*fimH*
N176 P3Mb	R	c	Musanze	*E. coli*	B1		*bla*_CTX-M1/15_, *bla*_TEM_, *tet*(A), *tet*(B), *qnrS*, *sul2*, *dfrA16*	*fimH*
N180 P7MDb	D	g	Musanze	*E. coli*	A	TOB, CHL, SXT	*bla*_CTX-M1/15_, *bla*_OXA-1_, *tet*(A), *tet*(B), *qnrS*, *sul2*, *dfrA17*	*fimH*, *papC*, *iucD*
N180 P7Ma	R	g	Musanze	*E. coli*	B1		*bla*_CTX-M1/15_, *qnrS*	*fimH*
N177 P4Ma	N	d	Musanze	*E. coli*	B1	TET, SXT	*bla*_CTX-M1/15_, *bla*_TEM_, *tet*(A), *qnrS*, *sul2*, *dfrA5*, *dfrA14*, *dfrA17*	*fimH*
N177 P4Mb	R	d	Musanze	*E. coli*	A	TET, SXT	*bla*_CTX-M1/15_, *bla*_TEM_, *tet*(B), *aadA4*, *sul1*, *sul2*, *dfrA17*	*fimH*
N177 P4MDb	M	d	Musanze	*E. coli*	A	SXT	*bla*_CTX-M1/15_, *bla*_TEM_, *tet*(B), *qnrS*, *sul2*, *dfrA14*	*fimH*
N178 P5Mb	R	e	Musanze	*E. coli*	A	TET	*tet*(A), *tet*(B), *bla*_CTX-M9_, *qnrS*, *dfrA5*	*fimH*
N179 P6Ma	N	f	Musanze	*E. coli*	A	SXT	*bla*_CTX-M9_, *bla*_CMY_, *aadA2*, *sul1*, *sul2*, *dfrA12*, *dfrA13*	*fimH*
N179 P6Mb	R	f	Musanze	*E. coli*	A	TET	*bla*_CTX-M1/15_, *tet*(A), *qnrS*, *dfrA14*	*fimH*
N179 P6MDb	R	f	Musanze	*E. coli*	B1	TET	*bla*_CTX-M1/15_, *bla*_ACT_, *tet*(A), *qnrS*	*fimH*
N185 P12Ma	R	l	Musanze	*E. coli*	A	TET, SXT	*bla*_CTX-M1/15_, *tet*(A), *qnrS*, *dfrA14*	*fimH*
N187 P14Ma	R	n	Musanze	*E. coli*	A	TET, SXT	*bla*_CTX-M1/15_, *bla*_TEM_, *tet*(A), *qnrS*, *sul2*, *dfrA14*	*fimH*
N187 P14Mc	M	n	Musanze	*E. coli*	B1	TET, SXT	*bla*_CTX-M1/15_, *bla*_TEM_, *tet*(A), *qnrS*, *sul2*, *dfrA14*	*fimH*
N188 P15Ma	R	o	Musanze	*E. coli*	A	TET, SXT	*bla*_CTX-M1/15_, *bla*_TEM_, *tet*(A), *aadA1*, *aadA2*, *qnrS*, *sul2*, *dfrA12*, *dfrA14*	*fimH*
N180 P7 Manure	M	g	Musanze	*E. coli*	A	TET, SXT	*bla*_CTX-M1/15_, *bla*_TEM_, *tet*(A), *aadA1*, *aadA2*, *qnrS*, *sul2*, *dfrA12*, *dfrA14*	*fimH*
N198 P25Na	R	dd	Nyagatare	*E. coli*	A	TET, SXT	*bla*_CTX-M1/15_, *bla*_TEM_, *tet*(A), *qnrS*, *sul2*, *dfrA14*	*fimH*
N199 P26Na	R	ee	Nyagatare	*E. coli*	A	TET	*bla*_CTX-M1/15_, *tet*(A), *qnrS*, *dfrA14*	*fimH*
N193 P20Ba	R	v	Bugesera	*E. coli*	A	SXT	*bla*_CTX-M1/15_, *bla*_TEM_, *qnrS*, *sul2*, *dfrA14*	*fimH*
1D	R	hh	Rwamagana	*E. coli*	B1	TET	*bla*_CTX-M1/15_, *tet*(A), *tet*(B), *qnrS*	*fimH*
2D	R	hh	Rwamagana	*E. coli*	A	TET, SXT	*bla*_CTX-M1/15_, *bla*_TEM_, *tet*(A), *aadA2*, *qnrS*, *sul1*, *sul2*, *dfrA12*, *dfrA14*	*fimH*
4D	R	hh	Rwamagana	*E. coli*	B1	TET, SXT	*bla*_CTX-M1/15_, *bla*_TEM_, *tet*(A), *qnrS*, *sul2*, *dfrA14*	*fimH*
6D	R	hh	Rwamagana	*E. coli*	B1	TET, CHL, SXT	*bla*_CTX-M1/15_, *bla*_TEM_, *tet*(A), *qnrS*, *sul2*, *dfrA14*	*fimH*
7D	R	hh	Rwamagana	*E. coli*	B1	TET, CHL, SXT	*bla*_CTX-M1/15_, *bla*_TEM_, *bla*_ACT_, *tet*(A), *qnrS*, *sul2*, *dfrA14*	*fimH*
9D	R	hh	Rwamagana	*E. coli*	B1	TET, SXT	*bla*_CTX-M1/15_, *tet*(A), *aadA4*, *qnrS*, *sul1*, *sul2*, *dfrA5*, *dfrA17*, *dfrA19*	*fimH*
10D	R	hh	Rwamagana	*E. coli*	A	TET, SXT	*bla*_CTX-M1/15_, *bla*_TEM_, *tet*(A), *qrnS*, *sul2*, *dfrA14*	*fimH*, *eae*, *acfC*, *esc*, *espL*
N176 P3MDmanure	M	c	Musanze	*E. coli*	A	CIP, TET, CHL, SXT	*bla*_CTX-M1/15_, *bla*_TEM_, *tet*(A), *sul2*, *dfrA14*	*fimH*, *papC*, *iucD*, *astA*
N195 P22Na	N	aa	Nyagatare	*K. pneumoniae*	NA	TET, SXT	*bla*_CTX-M1/15_, *bla*_SHV_, *bla*_TEM_, *tet*(A), *qrnB*, *qnrS*, *sul2*, *dfrA14*	NA
N195 P22Nb	R	aa	Nyagatare	*K. pneumoniae*	NA	TET, SXT	*bla*_CTX-M1/15_, *bla*_SHV_, *bla*_TEM_, *tet*(A), *qrnB*, *qnrS*, *sul2*, *dfrA14*	NA
N195 P22N Manure	M	aa	Nyagatare	*K. pneumoniae*	NA	TET, SXT	*bla*_CTX-M1/15_, *bla*_SHV_, *bla*_TEM_, *tet*(A), *qrnB*, *qnrS*, *sul2*, *dfrA14*	NA
N196 P23Na	R	bb	Nyagatare	*K. pneumoniae*	NA	TET, SXT	*bla*_CTX-M1/15_, *bla*_SHV_, *bla*_TEM_, *tet*(A), *qrnB*, *qnrS*, *sul2*, *dfrA14*	NA

* N = nasal, D = dust, R = rectal, M = manure. ** PG = *E. coli* phylogroup. *** TET, tetracycline; SXT, trimethoprim–sulfamethoxazole; CHL, chloramphenicol, CIP, ciprofloxacin; TOB, tobramycin. **** *E. coli* virulence-associated gene.

**Table 2:** Summary of the main phenotypic and genotypic characteristics of MRSA isolates investigated.

**MRSA Isolates**		N194P21Nasal	N194P21Dust	N195P22Manure	N195P22Dust
**Farm**		z	z	aa	aa
**District**		Bugesera	Bugesera	Nyagatare	Nyagatare
spa *		t011			
SCC*mec* **		IV			
CC ***		CC398			
**AMR profile**	Phenotype ****	β-lactams, GEN, TET			
	Genes detected	*blaZ*, *mecA*, *aacA-aphD*, *tet*(M)			
***cap* gene (*cap* 8)**		negative			
***cap* gene (*cap* 5)**		positive			
**Hemolysins**		*hla*, *hlb*, *hld*, *hlgA*			
**Leukocidins (luk) components**		*lukF*, *lukS*, *lukX*, *lukY*			
**Biofilm-associated genes**		*icaA*, *icaC*, *icaD*			
**Adhesion factors**		*clfA*, *clfB*, *cna*, *fnbA*, *fnbB*			

* *spa* = *spa* type, ** SCC*mec* = SCC*mec* type, *** CC = clonal complex, **** GEN = gentamicin, TET = tetracycline.

## Data Availability

The original contributions presented in this study are included in the article/Supplementary Materials. Further inquiries can be directed to the corresponding author(s).

## Supplementary Materials

The following supporting information can be downloaded at: https://www.mdpi.com/article/10.3390/ani16010122/s1, Table S1: Full hybridization results for MRSA isolates examined in this study; Table S2: Complete sample list.

## Abbreviations

The following abbreviations are used in this manuscript:

AMR	Antimicrobial resistance
WHO	World Health Organization
WHO BPP	WHO Bacterial Priority Pathogens List
3GC-R	Third-generation cephalosporin-resistant
MRSA	Methicillin-resistant *Staphylococcus aureus*
LA-MRSA	Livestock-associated MRSA
NVVH	New Vision Veterinary Hospital
MALDI-TOF MS	Matrix-assisted laser desorption ionization time-of-flight mass spectrometry
CLSI	Clinical & Laboratory Standards Institute
ExPEC	Extraintestinal pathogenic *E. coli*
UPEC	Uropathogenic *E. coli*
LEE	Locus of Enterocyte Effacement
EPEC	Enteropathogenic *E. coli*
EHEC	Enterohemorrhagic *E. col*
SCC*mec*	Staphylococcal Cassette Chromosome *mec*
ST	Sequence type
CC	Clonal complex
NAPAMR	National Action Plan on Antimicrobial Resistance
